# Bilateral inferior petrosal sinus sampling in the diagnosis of ACTH-dependent Cushing’s syndrome: experience in a tertiary hospital

**DOI:** 10.1515/almed-2022-0088

**Published:** 2022-10-03

**Authors:** Isabel Moreno Parro, David Ortiz Sánchez, Rosa García Moreno, Rubén Gómez Rioja, Remedios Frutos Martínez, Cristina Álvarez-Escolá

**Affiliations:** Laboratory Medicine Department, La Paz University Hospital-Carlos III-Cantoblanco, Madrid, Spain; Endocrinology Department, La Paz University Hospital-Carlos III-Cantoblanco, Madrid, Spain; Radiology Department, La Paz University Hospital-Carlos III-Cantoblanco, Madrid, Spain

**Keywords:** bilateral inferior petrosal sinus sampling, Cushing’s disease, Cushing’s syndrome, diagnostic utility study, ectopic ACTH syndrome

## Abstract

**Objectives:**

Bilateral inferior petrosal sinus sampling (BIPSS) is a useful test for differential diagnosis of central vs. ectopic adrenocorticotropic hormone (ACTH)-dependent Cushing’s syndrome (CS). We provide a description of the protocol used in our Center and an analysis of its diagnostic accuracy.

**Methods:**

A retrospective study was conducted of 28 patients who underwent BIPSS combined with corticotropin-releasing hormone (CRH) stimulation. The procedure is performed in an interventional neuroradiology suite, involving a multidisciplinary team of neuroradiologists, endocrinologists and laboratory professionals. The two petrosal sinuses are catheterized and a peripheral blood sample is obtained simultaneously, at baseline and at 3, 6 and 10 min following stimulation. ACTH and prolactin are determined by immunochemiluminescence.

**Results:**

A total of 19 cases of Cushing’s disease (CD) and 1 case of ectopic CS were confirmed. In all cases, BIPSS provided accurate diagnostic guidance, with a sensitivity and specificity of 100%. In 8 patients, remission was not achieved after surgery. In 84% of catheterizations, ACTH ratio peaked at 3–6 min following stimulation. Patients with histologically-confirmed CD exhibited higher sinus ACTH ratios and values. Prolactin ratio helped us identify and exclude 28.6% of the samples with inconsistent results.

**Conclusions:**

In our series, BIPSS combined with CRH stimulation demonstrated to be a safe, effective procedure. Prolactin emerges as a useful marker of correct catheterization. The participation of a multidisciplinary team is essential.

## Introduction

Cushing’s syndrome (CS) is a collection of clinical signs and symptoms resulting from prolonged exposure to high concentrations of glucocorticoids. The most frequent manifestations include weight gain, high blood pressure, and diabetes, with differential diagnosis playing a crucial role in these diseases. Other more specific manifestations include myopathy, skin atrophy, frequent hematoma, mental disorders, and hirsutism [1, 2].

The main cause of this condition is exogenous administration of glucocorticoids [1]. However, endogenous CS is clinically relevant and we can difference between those which shows elevated plasma levels of adrenocorticotropic hormone (ACTH) and those with normal levels [3]. The most common cause of ACTH-independent CS is overproduction of glucocorticoids by the adrenal glands due to the presence of an adenoma or carcinoma. ACTH-dependent CS may be due to a central cause, mainly as a result of a pituitary adenoma, or for an ectopic cause from non-pituitary ACTH-secreting tumors. When hypercortisolism is secondary to a tumor of the pituitary gland secreting ACTH, it is called Cushing’s disease (CD), which accounts for 60–80% of cases of endogenous CS [1]. The incidence of this entity in Europe is 0.7–2.4 cases per million population per year, being more prevalent in women than in men [4].

The origin of ACTH could be identified via corticotropin-releasing hormone (CRH)/desmopressin stimulation tests and the high-dose dexamethasone suppression test [5], combined with imaging tests, primarily magnetic resonance imaging (MRI). The two methods have significant limitations: the non-specificity of stimulation tests, the small size of microtumors and the possibility that non-functioning adenomas are identified. The treatment of choice in CD is trans-sphenoidal pituitary resection. Therefore, the use of tests with a high specificity is essential to avoid unnecessary surgery.

Bilateral inferior petrosal sinus sampling (BIPSS) was first performed in the ‘70s as a method to locate the origin of abnormal ACTH secretion. The first case of BIPSS in a patient with CS was reported by Corrigan et al. [6].

BIPSS is a diagnostic test that requires the intervention of a multidisciplinary team. It is requested and interpreted by endocrinologists, who control the withdrawal of adrenal suppression, common in these cases. BIPSS is performed by an interventionist radiologist and the samples are sent to the laboratory for subsequent processing. In this invasive study, a catheter is advanced via the femoral artery bilaterally into the petrosal sinuses. Additionally, a peripheral vein line is cannulated [3, 7]. Samples are simultaneously obtained from the three sites at baseline and following pharmacological stimulation of the pituitary gland at different time points within a 15-min period [3, 8], [9], [10]. Stimulation is generally performed using CRH or desmopressin, whose use has been proven to increase the sensitivity and specificity of the test [7]. Due to its high diagnostic value, BIPSS is the gold standard method for differentiating CD from ectopic CS [11].

The rationale for this test is that, in the presence of CD, ACTH concentrations will be significantly higher in blood from the petrosal sinuses, as compared to peripheral blood [12]. Sinus/peripheral blood ACTH ratios were used to facilitate the interpretation of differences in ACTH concentrations. According to Oldfield et al. a ratio ≥2 at baseline or a ratio ≥3 after stimulation is suggestive of CD, whereas lower ratios are consistent with ectopic CS [13]. These cut-off values are still used in clinical practice.

Type II errors (patients with CD and catheterization result not suggestive of ACTH-secreting adenoma) may occur as a result of incorrect catheterization or the presence of anatomical vascular variants altering normal pituitary drainage. To overcome this challenge, prolactin has been suggested as a marker of correct catheterization [11] based on the selectivity index (SI), defined as sinus/peripheral prolactin ratio [14].

BIPSS is used in patients with CS which hypercortisolemia has been demonstrated to be ACTH-dependent to establish a differential diagnosis of CD vs. ectopic CS. Its use is especially indicated when the results of non-invasive functional tests are inconsistent, when adenoma is excluded by imaging studies, or in the presence of an adenoma <6 mm [2, 3, 15, 16]. This procedure involves some risks for the patient, such as malaise and post-catheterization groin hematoma, which occur in 1–4% of patients and, more rarely, thrombotic events [1, 3].

BIPSS has also been suggested to be useful to locate the adenoma within the pituitary gland and guide surgical examination. Lateralization is determined by calculating the intersinus ACTH gradient (right/left petrosal sinus ACTH ratio, or vice versa), with a ratio ≥1.4 being considered predictive of lateralization [9, 12].

In our hospital, the use of BIPSS combined with CRH stimulation was incorporated to common practice in 2001. In 2014, prolactin determination was included in the protocol. We present a case series study of patients seen in our Center and provide an analysis of diagnostic accuracy.

## Materials and methods

A retrospective study of patients undergoing BIPSS in La Paz University Hospital, Madrid, Spain, between 2001 and 2020.

In BIPSS, a catheter is advanced via the femoral artery bilaterally into the petrosal sinuses, additionally, a peripheral line is cannulated. The procedure is carried out by two experienced interventionist radiologists in an interventional neuroradiology suite of the Radiology Department.

Samples are obtained from each site at baseline and at 3, 6, and 10 min after the administration of a bolus of 100 µg of intravenous CRH. The time points established in the protocol were modified in 2011. Initially, blood samples were drawn at baseline and at 6 and 10 min post-stimulation. Since 2011, a sample is also collected at 3 min post-stimulation.

The use of contrast media should be reduced as much as possible to prevent the risk of contamination. Thus, contrast should flush out adequately before sampling. In our case, the sample is obtained using two 5 mL-syringes. The first syringe is used for purging, then, 2–4 mL of blood is drawn.

To prevent mixing-up, sampling and aliquoting is performed at the operating room by laboratory specialists together with surgeon’s assistant. The samples obtained from each site at the different time points are collected into a serum separator tube for prolactin measurement, and into a tube containing EDTA 2K with separator gel for ACTH measurement. To prevent time- and temperature-dependent ACTH degradation [17], samples are refrigerated and transported to the laboratory, where they are centrifuged and processed short after collection. ACTH determination is performed on Immulite (Siemens Healthineers) analyzer, whereas prolactin was measured on Advia Centaur (Siemens Healthineers) until 2018, and thereafter on Atellica Solution (Siemens Healthineers). Detection is performed by Immunochemiluminescence.

Once ACTH values have been obtained, sinus/peripheral ACTH ratios are estimated. CD is considered when ratio ≥2 at baseline or ≥3 in post-stimulation samples. A higher ratio in one of the sinuses at any of the four time points confirms CD [3].

In 2011, the protocol was modified to incorporate prolactin as an indicator of correct catheterization. Since then, SI is required for a sinus/peripheral ACTH ratio to be considered assessable. Only the sinus samples with an SI ≥1.8 are considered informative.

Diagnosis of CD is confirmed by the presence of a histologically-confirmed ACTH-secreting adenoma and/or when symptoms disappear after surgery.

We assessed the efficacy of BIPSS and MRI in determining lateralization by taking the visualization of an adenoma during surgery or on histopathological analysis as the result of reference.

All data were obtained through standard care, in compliance with the tenets of the Declaration of Helsinki.

Statistical analysis was performed using R statistical software (version 4.0.2) both for plotting and for between-subject comparison using the Kruskal-Wallis test.

## Results

During the study period, 28 patients underwent a BIPSS, with a mean age of 43.36 ± 11.89 years, 24 women (85.7%) and 4 men (14.3%). None of the patients developed postoperative complications. The procedure was repeated in only one patient due to sampling difficulties.

Sinus/peripheral ACTH ratio exceeded the cut-off value in 26 of the 28 patients, which was consistent with CD. All underwent trans-sphenoidal resection. CD was confirmed in 19 cases, 11 of whom had an ACTH-secreting adenoma, whereas 8 complied with post-surgery healing. CD could not be confirmed in the remaining 7 patients (Table 1).

**Table 1:** Patient distribution according to ACTH ratio and post-surgery outcome.

BIPSS results Total n=28
Sinus/peripheral ACTH ratio ≥2 at baseline and/or ≥3 poststimulation=26
Confirmed CD 19
CD not confirmed 7
Sinus/peripheral ACTH ratio <2 at baseline and <3 poststimulation=2
Confirmed CD 1
CD not confirmed 1

The 2 other patients exhibited sinus/peripheral ACTH ratios below the cut-off, which was suggestive of an ectopic origin. The presence of an ACTH-secreting pulmonary carcinoid tumor was confirmed by immunohistochemistry in a patient. The other patient underwent a pulmonary lobectomy due to a suspicious image, although the presence of a tumor could not be confirmed on the surgical sample, and the patient remained symptomatic after surgery. In this case, incorrect catheterization could not be excluded, as it was performed prior to the incorporation of the SI to the protocol.

Considering the 20 patients with a confirmed diagnosis, BIPSS showed a sensitivity and specificity of 100% for the diagnosis of CD or ectopic CS. These values were attained both at baseline and post-stimulation.

**Figure 1:**
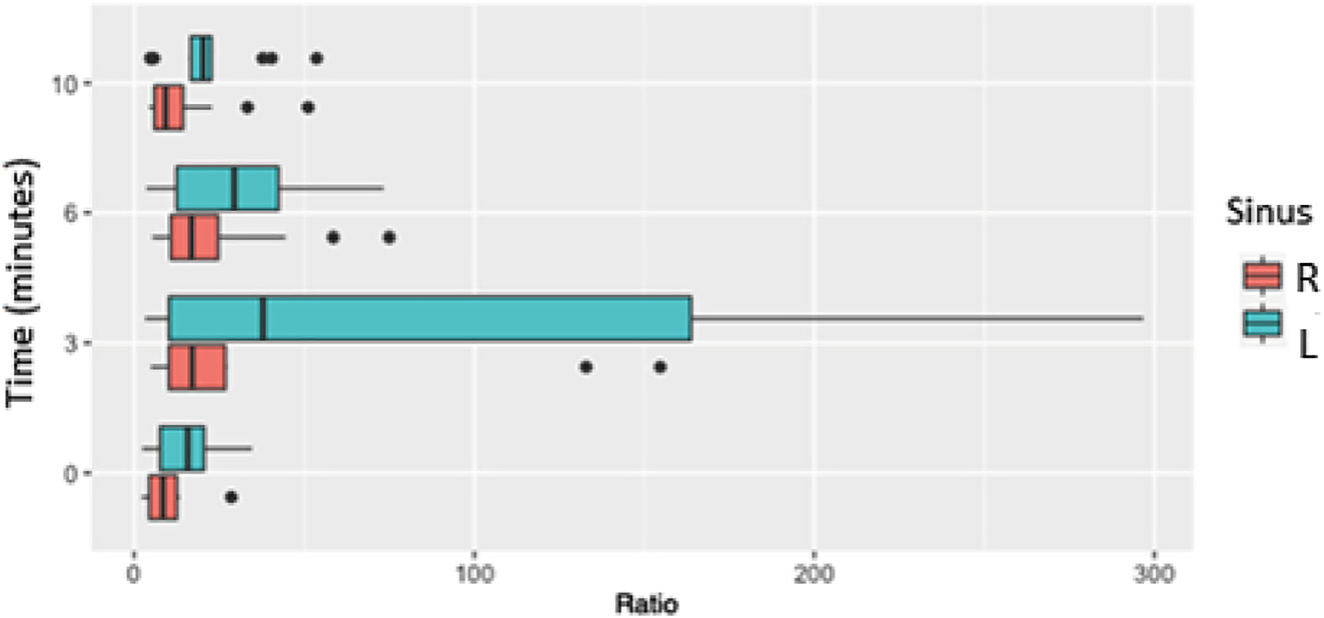
Sinus/peripheral ACTH ratios at the different time points of patients with confirmed CD.

To identify the time point with the highest ratios, the ratios obtained in patients with a confirmed diagnosis of CD were analyzed at the different time points. ACTH ratio peaked 3–6 min post-stimulation in 16 catheterizations (84%), at baseline in 1 (5%) and at 10 min post-stimulation in 2 cases (11%). Plotting of CD-suggestive ratios revealed that ratios peaked at 3 min post-stimulation (Figure 1).

**Figure 2:**
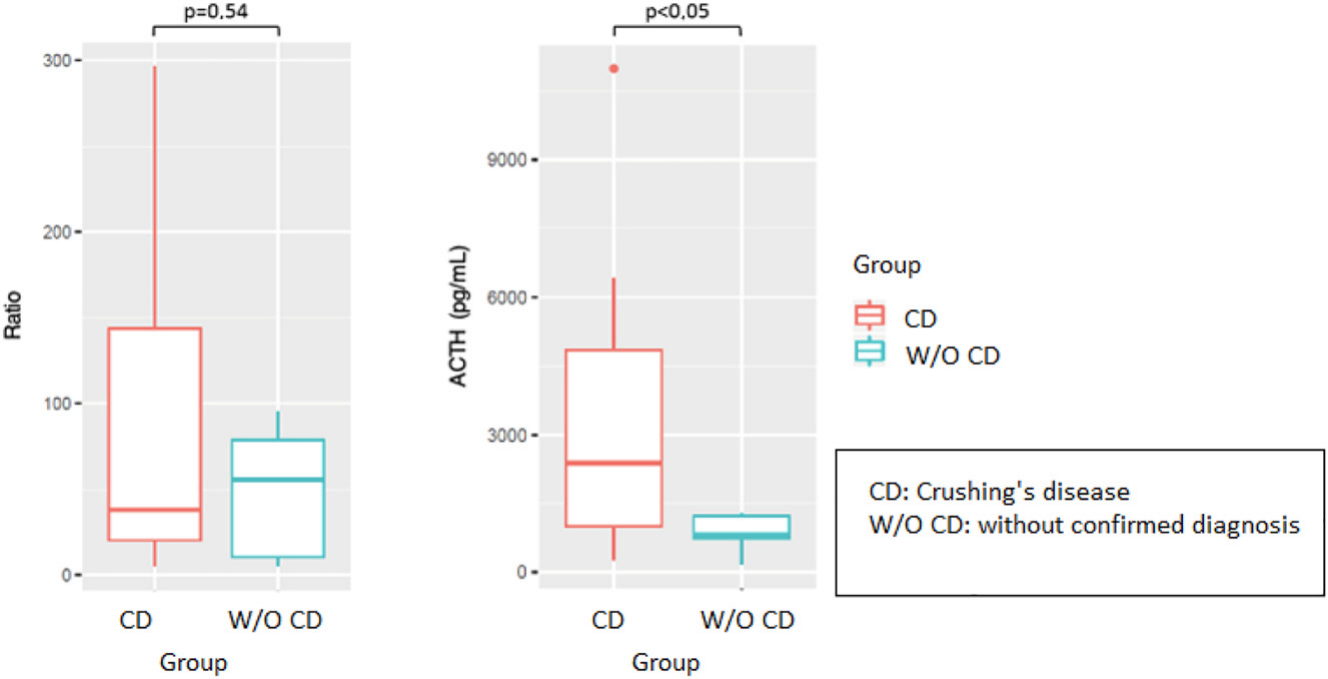
Differences in ACTH ratios and peak sinus ACTH values between the group of patients with CD and the group of patients without a confirmed diagnosis.

Considering all patients with BIPSS results consistent with CD, we analyzed potential differences in ACTH ratios and sinus ACTH levels between the group with confirmed CD and the group without a confirmed diagnosis. No statistically significant differences were observed in ACTH ratios between groups. However, ACTH values were significantly higher in the group of patients with CD (Figure 2).

Of all BIPSS, prolactin SI was used as an indicator of correct catheterization in 21 cases. In 7 catheterizations, SI was ≥1.8 in only one sinus whereas, in the remaining cases, catheterization was correct in the two sinuses in at least a time point. Considering all time points in the 21 patients, the sample was representative of pituitary drainage in the right sinus in 76.2% (64/84) of samples, and in the left sinus in 66.7% (56/84) of cases. The location of the catheter at sampling was adequate in 71.4% (120/168) of cases.

Of the 104 samples obtained from the 13 patients with confirmed CD where SI was calculated, SI was below the cut-off in 25, which suggests that the sample was not representative. The ACTH ratio was below the cut-off in most of these non-informative time points (16 time points in 6 patients). This was suggestive of an ectopic origin, which was inconsistent with other results in the study.

The lateralization results of BIPSS were analyzed considering the 17 cases in which conclusive results were obtained, with histopathological confirmation of an intrapituitary location of the tumor. The intersinus ACTH ratio ≥1.4 criteria were applied for the interpretation of lateralization. In two patients, there were discrepancies across the four time points; as a result, they were classified as undetermined. Concordance of intersinus ACTH ratio with histopathological analysis was 64.7% (Table 2), vs. 85.7% for MRI results, which were available in 14 cases.

**Table 2:** Concordance between the lateralization results obtained from BIPSS vs histopathological analysis after surgery.

		Lateralization based on BIPSS (inter-sinus ACTH ratio ≥1.4)		
		Right	Undetermined	Left
Lateralization observed by histopathological analysis	Right (9)	5 (56%)	2 (22%)	2 (22%)
	Left (8)	2 (25%)	0	6 (75%)

## Discussion

The diagnosis of CD may be challenging. Non-invasive tests are the initial methods used for determining ACTH-dependency and differentiating CD from ectopic CS. However, the results of these tests do not always provide solid evidence that surgery is indicated. It is in these cases where BIPSS emerges as a useful tool [18, 19]. In our study, the results of imaging studies were negative in 7 of the 28 patients who underwent BIPSS. Of the remaining 19, the size of adenoma was 10 mm in 1 case and ≤9 mm in the other cases, which is consistent with the recommendations of recent guidelines for the diagnosis and management of CD [11].

In our series, BIPSS was crucial in the diagnosis of a case of ectopic CS that had not been detected in previous studies. The highest sinus/peripheral ACTH ratios were 1.36 and 1.97 in the baseline and post-stimulation sample, respectively (which corresponded to sinus ACTH values of 139 and 187 pg/mL). It is worth noting that prolactin SI confirmed correct catheterization of the two sinuses at all time points.

In another patient with ACTH ratios suggestive of ectopic CS, subsequent studies revealed a possible pulmonary neoplasm, which could not be confirmed histologically. Moreover, total healing was not achieved after lobectomy. In this case, prolactin determination had not yet been incorporated to the protocol to confirm a pituitary origin of venous drainage.

In the 19 cases of confirmed and solved CD, the most informative sinus/peripheral ACTH ratio during the procedure ranged from 5.8 to 296.8, which corresponds with sinus ACTH concentrations from 327 to 10,980 pg/mL, respectively. In 84% of catheterizations, the ratio peaked between 3 and 6 min post-stimulation.

In a series of 470 cases of histologically-confirmed CD who underwent BIPSS [9], Wind et al. observed that ACTH ratios correlated strongly with sinus ACTH concentrations, and that ACTH <200 pg/mL at baseline and <400 pg/mL following CRH are related to false negatives for CD.

We performed a comparative analysis of petrosal sinus ACTH concentrations and ACTH ratios in the 19 patients with confirmed CD vs. the 7 patients in whom CD could not be confirmed. The results revealed no statistically significant differences between groups in ACTH ratios. Nevertheless, the group with confirmed CD exhibited significantly higher ACTH concentrations.

Symptoms did not disappear after pituitary surgery in 7 patients whose catheterization results were suggestive of CD. During follow-up of these patients, repeating surgery was not considered to be indicated, and CD could not be confirmed despite suspicion.

BIPSS had a diagnostic efficacy of 100% in the cases where all symptoms were confirmed to have disappeared after surgery, which is consistent with the criteria applied in previous studies, with a reported sensitivity of 85–100% and a specificity of 67–100% [3, 12]. However, in our series, 29% of cases could not be confirmed histologically or did not achieve healing. Similar results have been obtained in previous studies [19]. Occasionally, post-surgery remission of CS is not achieved, which occurs regardless of whether BIPSS is used or not in the series.

Getting one sample at baseline and three samples post-stimulation makes the procedure more difficult, since it requires that the catheter is maintained in an adequate position for longer. However, it also increases the chances of obtaining an informative sample. During the procedure, the use of contrast to determine the morphology of the venous drain system and ensure adequate catheter positioning should be limited, as it increases the need to purge the sample and involves patient irradiation. The handling of probes or mobilization of the patient during sampling may cause catheter displacement and cause blood from the pituitary glands to dilute in brain fluid. For this reason, most case studies recommend multiple sampling, generally between 3 and 15 min post-stimulation. In our study, we observed that ACTH secretion peaked 3–6 min following stimulation.

BIPSS is especially useful when imaging studies exclude pituitary adenoma. In this setting, false negatives, near 10% in most studies [7], have been associated with non-representative samples. The potential presence of anatomical variants in the vascular system of petrosal sinuses should be considered, since it results in abnormal pituitary drainage and makes catheter positioning more difficult [20, 21].

There are 6 types of variants, based on the way blood is drained from the inferior petrosal sinuses through the jugular vein [21]. These variants may be found either in one or the two sinuses. They may hamper correct access and unbalance pituitary hemisphere drainage, which may lead to SI misinterpretation and confusion in the diagnosis of lateralization.

An *a posteriori* way to assess the quality of the sample of pituitary blood is through simultaneous measurement of prolactin. Prolactin is a hormone secreted by the pituitary glands in regions that are generally apart from corticotroph cells, with a longer half-life in the circulation. An SI ≥1.8 identifies the blood samples most likely to have been drained from the pituitary glands, thereby increasing the specificity of BIPSS [22].

Some authors [14, 22] recommend the standardization of ACTH ratios with respect to prolactin ratios. Thus, a normalized ratio >0.8 would be suggestive of CD, although an adequate pituitary effluent may have not been obtained. Other publications [23, 24] suggest that this standardization could not be applicable to patients with ectopic CS and recommend the use of prolactin ratio only to identify the informative points of the test.

In our series, since its incorporation, SI determination identified 28.6% of the samples with suspicion of inadequate catheter positioning, which generally provided inconsistent results with other samples. In six patients with confirmed CD in one of the sinuses might have been interpreted as negative if SI had not been used. In the case of the patient with confirmed ectopic CS, SI confirmed that catheterization was correct and provided guidance for further examination for an ectopic origin.

Pre-surgery confirmation of lateralization emerges as a useful tool for a more conservative approach to be adopted, which would avoid total transsphenoidal surgery. In our series, we found a good correlation between lateralization confirmed by surgical localization and the lateralization obtained either biochemically or on imaging studies (64.7 and 85.7%, respectively). The results of biochemical lateralization in our series are in agreement with the literature [2, 9, 21].

BIPSS consistently emerges as a gold standard method for differential diagnosis of CD vs. ectopic CS [3, 8, 10, 12, 15]. It is an invasive test that requires specific equipment and a multidisciplinary team of specialists, but with high success rates and infrequent complications. Collaboration with the laboratory during BIPSS is essential to ensure the appropriate processing of extremely useful samples. Multiple-time post-stimulation sampling and prolactin measurement are recommended for more effective diagnostic testing.
